# Bilateral spontaneous massive renal hemorrhage in a peritoneal dialysis patient: A case report

**DOI:** 10.1097/MD.0000000000027549

**Published:** 2021-11-05

**Authors:** Tzu-Cheng Wen, Kuo-Hua Lin, Pin-Fang Chiu, Kuo-Sheng Lin, Chih-Wei Lee, Chien-Pin Chan

**Affiliations:** a Division of General Surgery, Changhua Christian Hospital, Changhua, Taiwan; b Division of Nephrology, Department of Internal Medicine, Changhua Christian Hospital, Changhua, Taiwan; c Surgical Intensive Critical Care Unit, Department of Emergency, Changhua Christian Hospital, Changhua, Taiwan; d Department of Radiology, Changhua Christian Hospital, Changhua, Taiwan.

**Keywords:** bilateral spontaneous massive renal hemorrhage, hemodialysis, peritoneal dialysis, transcatheter arterial embolization, Wünderlich syndrome

## Abstract

**Rationale:**

Non-traumatic bilateral spontaneous massive renal hemorrhage confined to the subcapsular and perirenal space, also known as Wünderlich syndrome, can occur suddenly and insidiously and cause serious consequences if not properly identified and managed. We report a case of bilateral spontaneous massive renal hemorrhage in a series of devastating episodes.

**Patient concerns:**

A 38-year-old woman undergoing peritoneal dialysis for 7 years for end-stage renal disease presented with disturbances in consciousness and sudden hypotension.

**Diagnosis:**

The patient's laboratory results indicated an abrupt drop in hemoglobin level. Emergent abdominal computed tomography (CT) showed a rupture of the lower pore of the left kidney, with massive hemoretroperitoneum. A second sudden reduction in hemoglobin level occurred 2 months later during the same admission course, with poor response to urgent blood transfusion. Contrast extravasation at the lower pole of the right kidney and posterior pararenal space along with a subcapsular hematoma was revealed on abdominal CT.

**Intervention:**

The patient's initial episode was managed with emergent transcatheter arterial embolization (TAE) of the left renal artery and again after the second episode for occlusion of the inferior branches of the right renal artery.

**Outcomes:**

After the first episode, immediate postprocedural angiography showed total occlusion of the left renal artery without contrast extravasation. Follow-up CT performed 10 days after the first TAE showed a residual left perirenal hematoma that extended to the left retroperitoneal and left upper pelvic region, without active bleeding. No follow-up imaging was done after the second TAE except for immediate postprocedural angiography, which showed no additional contrast extravasation of the right renal artery.

**Lessons:**

Bilateral spontaneous massive renal hemorrhage is rare and generally occurs in patients undergoing dialysis. Known studies appear primarily in case reports. Most patients can be treated successfully with TAE when diagnosed early.

## 1 Introduction

Non-traumatic spontaneous kidney bleeding confined to the subcapsular and perirenal space, also known as Wünderlich syndrome, can occur suddenly and insidiously and cause serious adverse effects if not properly identified and treated.^[1]^ Historically, clinical signs known as Lenk triad, consisting of acute unilateral flank pain, palpable lumbar mass, and general malaise with hypovolemic shock,^[2]^ have been identified as symptoms of the disease. However, all 3 symptoms were seen in only approximately 20% of patients.^[3]^ Upon diagnosis of bilateral spontaneous massive renal hemorrhage, management is rather simple. The current primary treatment is transcatheter arterial embolization (TAE) or curative nephrectomy if embolization fails. In this study, we report the case of a patient with bilateral spontaneous massive renal hemorrhage, which was successfully treated with TAE at 2 different time points in 1 single admission course.

### 1.1 Case report

We present a 38-year-old woman who began peritoneal dialysis in November 2012 for chronic glomerulonephritis caused by ovulation medication and subsequent renal failure. She also had polycystic kidney disease. Due to encapsulated sclerosing peritonitis with small bowel obstruction, she switched from peritoneal dialysis to hemodialysis in August 2019.

The patient developed disturbances in consciousness within days after the starting regular hemodialysis. Her Glasgow Coma Score decreased from 14 (E4M6V4) to 9 (E3M4V2), and her clinical presentation included drowsiness, cold sweating, and pale conjunctiva. Vital signs were temperature, 37°C; heart rate, 102 beats per minute; respiratory rate, 32 breaths per minute; and blood pressure, 89/66 mm Hg. Oxygen saturation in room air was 100%. A bedside abdominal physical examination found distension and muscle guarding, with prominent peritoneal signs. Blood analysis showed prominent leukocytosis (21,500 cells/μL) with predominant neutrophils of 86.5%. There was an abrupt drop in hemoglobin level, from 8.5 to 6.5 g/dL. Arterial blood gas analysis reported prominent hypoxia (PaO_2_ = 52.4 mm Hg) and mild respiratory alkalosis with CO_2_ washout (pH = 7.485, PCO_2_ = 31.6 mm Hg, and HCO_3_ = 24.0 mmol/L). Platelet count, prothrombin time, and activated partial thromboplastin time were all normal. Blood chemistry tests showed elevated C-reactive protein at 2.28 mg/dL, poor renal function (compatible with a patient undergoing dialysis), and normal electrolyte concentration. Our clinical conclusions were an intra-abdominal hollow organ perforation initially and intra-abdominal bleeding secondarily.

**Figure 1:**
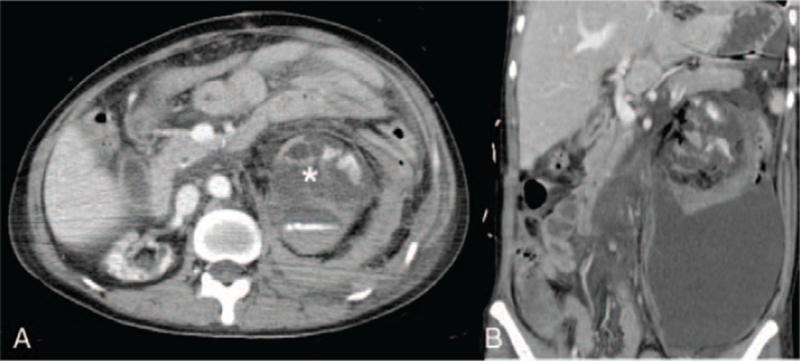
(A) Contrast extravasation of the left renal artery in computed tomography (transverse section). Asterisk: Contrast extravasation of the left renal artery. (B) Contrast extravasation of the left renal artery with massive left renal hematoma in computed tomography (coronal section).

**Figure 2:**
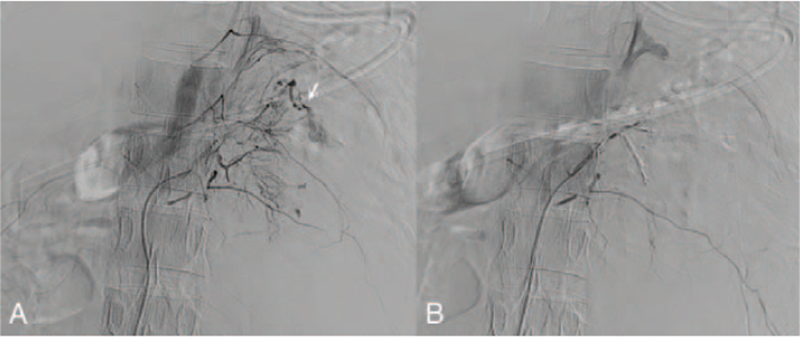
(A) Angiography before transarterial embolization. Arrow: Active extravasation of the left renal artery on angiography. (B) No contrast extravasation after transarterial embolization of the left renal artery.

Emergent abdominal computed tomography (CT) showed a rupture of the lower pore of the left kidney with massive hemoretroperitoneum (Fig. 1). TAE of the left renal artery was performed (Fig. 2A). Postprocedural angiography showed total occlusion of the left renal artery without contrast extravasation (Fig. 2B). Follow-up CT performed 10 days later showed a persistent left perirenal hematoma without active bleeding.

**Figure 3:**
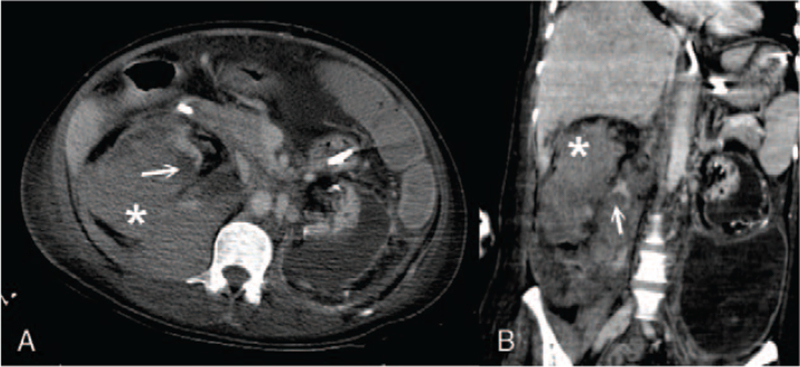
(A) Contrast extravasation of the right renal artery in computed tomography (transverse section). Arrow: Contrast extravasation of the right renal artery. Asterisk: Massive right renal hematoma. (B) Contrast extravasation of the right renal artery with massive right renal hematoma in computed tomography (coronal section). Arrow: Contrast extravasation of the right renal artery. Asterisk: Massive right renal hematoma.

**Figure 4:**
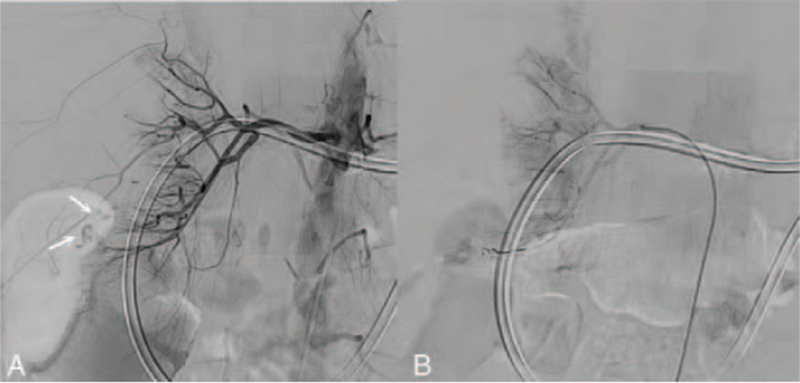
(A) Contrast extravasation of the right renal artery on angiography. Arrows: Extravasation of contrast medium noted from inferior branches of the right renal artery. (B) Absence of extravasation after transcatheter arterial embolization of the right renal artery.

A second sudden onset of change in consciousness occurred 2 months later, accompanied by tachypnea, during the same admission course. The patient's Glasgow Coma Score decreased from 14 (E4M6V4) to 11 (E4M4V3). Her vital signs were temperature, 38.2°C; heart rate, 122 beats per minute; respiratory rate, 33 breaths per minute; and blood pressure, 118/61 mm Hg. Oxygen saturation was 94% under 3 liters per minute of oxygen support. She experienced cold sweating with chills without prominent abdominal tenderness or peritoneal signs during this second episode. We considered recurrent intra-abdominal infection or a new episode of left renal spontaneous bleeding. During this second episode, blood analysis showed an abrupt drop in hemoglobin level, from 10 to 4 g/dL. The patient had mild leukocytosis (10,000 cells/μL) with predominant neutrophils of 92.5%. A coagulation profile showed mild thrombocytopenia (123,000 cells/μL), a prolonged prothrombin time (13.7 seconds), and a prolonged activated partial thromboplastin time (40.7 seconds). Blood chemistry tests showed hyperkalemia (5.4 mEq/L), hypercalcemia (2.64 mmol/L), and hyperphosphatemia (1.77 mmol/L), which were compatible with a pre-dialysis electrolyte profile. The patient's hemoglobin level did not improve after blood transfusion of 2 units of packed red blood cells. Contrast extravasation at the lower pole of the right kidney and posterior pararenal space along with a subcapsular hematoma was shown on abdominal CT (Fig. 3). TAE was performed again for occlusion of the inferior branches of the right renal artery (Fig. 4A). Postprocedural angiography of the right renal artery showed no contrast extravasation (Fig. 4B). Hypoxia soon developed, and the patient died from acute respiratory failure.

## 2 Discussion

Bilateral spontaneous massive renal hemorrhage was first described by the German physician Carl Reinhold August Wünderlich in 1856.^[4]^ The etiology of bilateral spontaneous massive renal hemorrhage was primarily renal benign neoplasm with angiomyolipoma as the predominant tumor, followed by renal cell carcinoma. A 2002 meta-analysis of the etiology of spontaneous perirenal hemorrhage in 165 patients showed a similar distribution of causes. Statistics indicated half of the causative renal neoplasms were malignant.^[5]^ Other common etiologies have been described, including tuberous sclerosis, vascular lesions (e.g., polyarteritis nodosa), arteriovenous malformations, renal artery aneurysms, ruptured renal cysts, renal calculi, and coagulopathy. Non-traumatic extrarenal causes of spontaneous retroperitoneal hematoma include ruptured abdominal aortic aneurysms and other retroperitoneal-originated pathologies.^[3,5]^

Factors for high risk of spontaneous renal rupture in patients with renal tumors include a tumor larger than 4 cm,^[3]^ aspirin and/or warfarin or other antiplatelet treatment,^[6]^ and hemodialysis rather than peritoneal dialysis treatment.^[7,8]^ Acquired cystic kidney disease could be a potential risk factor as well because the most common complication of acquired renal cysts is a hemorrhage confined within cysts.^[9]^ Larger cysts cause a wide fluctuation of intra-cystic pressure, and subsequent alterations in the dynamics of the cystic fluids may have contributed to a higher risk of spontaneous cystic rupture.^[10]^ Moreover, our patient had an acquired cystic kidney disease and switched from peritoneal dialysis to hemodialysis. Considering the abovementioned multiple risk factors for spontaneous renal rupture, this possibly explains her death following 2 serial spontaneous renal hemorrhages.

Historically, clinical signs of Lenk triad were identified as symptoms of the disease. However, all 3 symptoms were seen in only approximately 20% of cases.^[3]^ Our patient experienced the first 2 (acute unilateral flank pain and palpable lumbar mass), and disease progression was identified quickly in both episodes. Despite the small chance that all 3 symptoms would appear, it is critical to consider this diagnosis when treating a patient on dialysis with acute abdominal pain and a sudden decrease in hemoglobin.^[6]^

Upon diagnosis of bilateral spontaneous massive renal hemorrhage, management is rather simple. Discontinuation of anticoagulants and replacement of blood volume with close monitoring of vital signs and hemoglobin levels are sensible first-line treatments for patients who are hemodynamically stable.^[7]^ With the improvement in non-invasive vascular procedures, the current primary treatment for unstable patients is TAE or curative nephrectomy if embolization fails.^[11,12]^ Embolization procedures for renal vascular lesions performed with platinum microcoils had shown successful outcomes without major complications, such as large renal parenchymal infarction or iatrogenic morbidity.^[13]^ In 2018, Xie et al^[8]^ reported 3 patients with spontaneous perirenal hemorrhage who were treated successfully with gelatin sponge embolization.

Surgical exploration and curative nephrectomy are strongly suggested if embolization fails.^[7]^ We made a rapid diagnosis during both episodes experienced by our patient. We followed the current evidence in treating bilateral spontaneous massive renal hemorrhage, with satisfactory results because there was no further extravasation, and the patient's bleeding stabilized. However, we noted a retained massive perirenal hematoma soon after the first TAE and suspected that it caused the patient's intermittent episodes of fever. We performed multiple sonography-guided needle aspiration of the hematoma, yet her fever was not resolved entirely. This raises clinical concerns about unresolved perirenal hematoma after TAE; therefore, further investigation of subsequent treatment options in such conditions may be required since there are no strong evidences regarding it in the literature.

In conclusion, Wünderlich syndrome is a rare but critical condition, especially in high-risk patients with renal tumor or coagulopathy. Bilateral events should be prevented for high-risk patients. Further, TAE was proven to be effective for the treatment of active bleeding in patients with Wünderlich syndrome, yet curative nephrectomy may be necessary if embolization fails.

## Author contributions

**Conceptualization:** Chih-Wei Lee.

**Investigation:** Tzu-Cheng Wen.

**Supervision:** Kuo-Hua Lin, Pin-Fang Chiu, Kuo-Sheng Lin, Chih-Wei Lee, Chien-Pin Chan.

**Writing – original draft:** Tzu-Cheng Wen.

**Writing – review & editing:** Kuo-Hua Lin.
